# Le mélanome primitif de la muqueuse génitale féminine: à propos de trois observations et revue de littérature

**DOI:** 10.11604/pamj.2013.16.58.2404

**Published:** 2013-10-19

**Authors:** Amal Bennani, Hinde el Fatemi, Sanae Erraghay, Hind Mobakir, Hassania Ameurtess, Ihsane Souuaf, Kaoutar Moumna, Karima Idrissi, Asmae Zriouel, Nadia Squalli, Abdelaziz Banani, Affaf Amarti

**Affiliations:** 1 Service d'anatomie pathologique CHU Hassan II Fès, Maroc; 2 Service de gynécologie obstétrique CHU Hassan II Fès; 3 Service de radiologie CHU Hassan II, Fès, Maroc

**Keywords:** Mélanome, muqueuse génitale, col utérin, métrorragies, Melanoma, genital mucosa, cervix, metrorrhagia

## Abstract

Le mélanome malin primitif de l'appareil génital féminin est une tumeur extrêmement rare. Il est fréquemment observé au niveau de la vulve mais il est rare au niveau du col utérin et du vagin. Il est le plus souvent diagnostiqué à un stade tardif à l'occasion de métrorragies ou de masse tumorale. Son histogénèse a été longtemps débattue. Le diagnostic est anatomo-pathologique avec recours nécessaire à l'étude immunohistochimique. Sa prise en charge n'est pas codifiée avec plusieurs thérapeutiques proposées notamment dans le mélanome métastatique. Son pronostic est désastreux, associé à un taux élevé de récidives et à une courte survie. Les auteurs présentent trois observations, de mélanomes primitifs vaginal, vulvaire et cervical, chez trois patientes âgées respectivement de 70, 65 et 40 ans. Et à travers ces observations, ils mettent en relief les principaux aspects cliniques, histologiques, thérapeutiques de cette entité avec une revue de la littérature.

## Introduction

Le mélanome primitif de l'appareil génital féminin est une tumeur extrêmement rare et représente moins de 2% de l'ensemble des mélanomes. De ce fait le diagnostic est souvent initialement méconnu et se fait à un stade tardif. Son évolution est défavorable avec des métastases viscérales fréquentes et une survie courte. Les auteurs rapportent trois observations de mélanome primitif de l'appareil génital féminin avec une revue de la littérature.

## Patients et observations

### Observation 1

**Figure 1:**
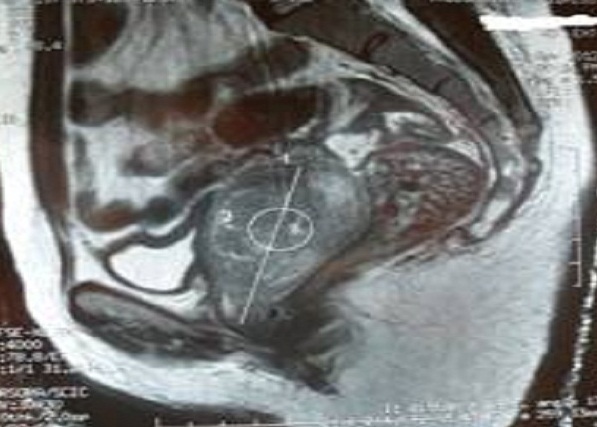
Coupe sagittale pondérée T1 montrant une masse vaginale en continuité avec le col utérin (observation 1)

**Figure 2:**
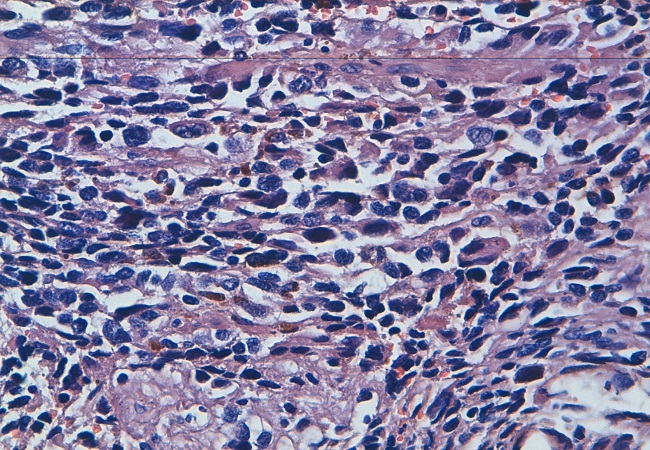
Prolifération indifférenciée faite de cellules disposées en nappes diffuses comportant du pigment brunâtre en intra-cytoplasmique avec des atypies cyto-nucléaires manifestes à fort grossissement (HESx40)

Nous rapportons le cas d'une femme de 40 ans, célibataire, nulligeste, qui consulte pour des métrorragies de moyenne abondance remontant à 3 mois. Le toucher rectal met en évidence une masse irrégulière mal limitée de consistance ferme comblant le cul de sac de Douglas. L'échographie pelvienne a permis d'objectiver une masse tissulaire échogène retro-utérine de 60 x 45cm en continuité avec l'utérus. L'IRM a mis en évidence un volumineux polype endovaginal prenant naissance au niveau du col avec extension paramétriale proximale gauche et contact intime avec la paroi ano-rectale (Figure 1). Un bistournage de la masse a été réalisé. L'analyse histologique et immunohistochimique était en faveur d'un mélanome exprimant le CD117 (Figure 2). Le bilan d'extension comprenait une TDM thoraco-abdomino-pelvienne, un examen dermatologique complet et un examen ophtalmologique. Il était négatif permettant ainsi de retenir le diagnostic d'un mélanome primitif du col utérin. La TDM thoraco-abdomino-pelvienne a objectivé des micro-nodules pulmonaires dont la nature métastatique est incertaine avec une thrombose veineuse iliaque droite. La décision était de mettre la malade sous anticoagulant avec une chimiothérapie palliative à base de Dacarbazine.

### Observation 2

**Figure 3:**
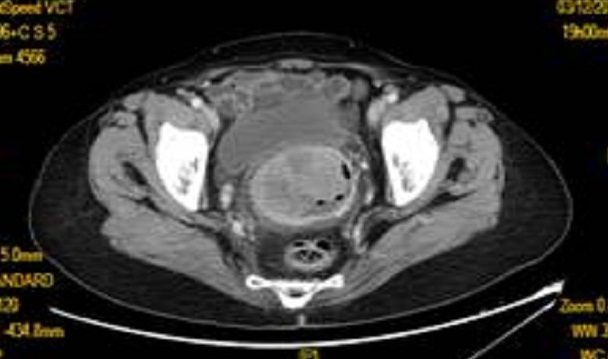
Coupe transversale C+ montrant une masse vaginale se rehaussant de manière hétérogène après injection du produit de contrast et ayant un contact intime avec la paroi postérieure vésicale (observation 2)

Il s'agit d'une patiente âgée de 70 ans, mariée et mère de 6 enfants, ménopausée il y a 30 ans, qui s'est présentée avec des métrorragies post ménopausiques depuis 6mois, des leucorrhées ainsi qu'une sensation de tuméfaction vaginale, le tout évoluant dans un contexte d'amaigrissement et d'altération de l'état général. L'examen gynécologique a retrouvé une formation tumorale implantée sur la face antérieure du vagin mesurant 3cm de grand axe, étendu du tiers moyen jusqu'à le cul de sac postérieur et englobant le col. Une biopsie de cette masse a été réalisée. Le diagnostic anatomopathologique était celui d'un mélanome vaginal exprimant le CD 117 à l'étude immunihistochimique.L'examen dermatologique, ainsi que l'examen ophtalmologique étaient sans particularités. Une TDM Thoraco-abdomino-pelvienne a montré un processus bourgeonnant occupant toute la cavité vaginale se rehaussant de manière hétérogène après injection du produit de contrast avec importante infiltration de la graisse péri-vaginale (Figure 3). Ce processus s'associait à de multiples adénopathies péri lésionnelles et iliaques infra-centimétriques ainsi qu'à de multiples micronodules pulmonaires diffus dont l'aspect évoque des métastases. La malade a été mise sous IMATINIB avec régression de la masse et des micro-nodules pulmonaires après trois cures puis décès après 1 an et demi du diagnostic.

### Observation 3

**Figure 4:**
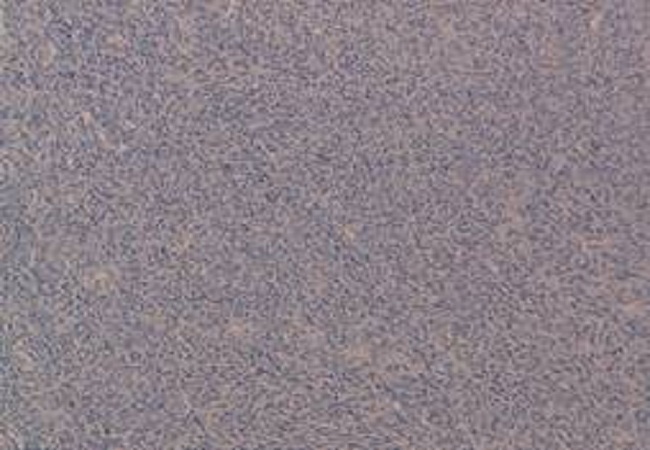
prolifération fusocellulaire dense (observation 3)

Une patiente âgée de 65 ans, G8P9, ménopausée il ya 10 ans. Cette patiente a comme antécédents chirurgicaux une hystérectomie totale pour des métrorragies post ménopausiques. L'examen anatomopathologique de la pièce était sans particularité. L'évolution a été marquée par l'apparition d'adénopathies inguinales de 1 à 2cm de grand axe. L'examen clinique a retrouvé une tumeur vulvaire péri-méatique mesurant 2cm de grand axe, d'aspect blanchâtre ferme. La biopsie de cette masse a montré une prolifération fusocellulaire (Figure 4) faite de cellules atypiques exprimaient fortement l'anticorps anti Melan A, anti PS100. Le CD117 était négatif. La malade a bénéficié d'une TDM thoraco-abdomino-pelvienne qui a mis en évidence de multiples micro-nodules pulmonaires suspects de localisations secondaires ainsi que des adénopathies inguinales bilatérales. La patiente a bénéficié d'une vulvectomie avec curage ganglionnaire inguinale bilatérale. L'examen anatomopathologique de la pièce a montré la même prolifération retrouvée sur la biopsie. Les ganglions étaient tous métastatiques. La décision thérapeutique é été de mettre la patiente sous chimiothérapie palliative à type de Dacarbazine.

## Discussion

Le mélanome malin des muqueuses est une tumeur extrêmement rare. Il représente 0,0 3% de l'ensemble des cancers [1]. Il survient dans différentes localisations notamment la cavité orale, l'anus, la conjonctive et plus rarement au niveau de la muqueuse génitale féminine. Cette dernière localisation représente moins de 2% de l'ensemble des mélanomes [1]. Dans ce cas, il survient au niveau du vagin et de la vulve, plus rarement au niveau du col utérin. Les mélanocytes proviennent embryologiquement de la crête neurale. Lors de sa migration vers l'épiderme, certains mélanocytes peuvent rester de façon aberrante dans la muqueuse vaginale ou dans le canal endocervical. Ils seront alors à l'origine de mélanomes primitifs vaginaux, cervicaux ou vulvaires. Contrairement aux mélanomes cutanés, il n'a pas été mis en évidence de facteurs de risque génétiques prédisposant à la survenue de ces tumeurs [2]. Le rôle du facteur hormonal dans le développement de ces mélanomes, a été évoqué par KHOO et al [3]. L'analyse des cas de mélanomes primitifs vaginaux et cervicaux rapportés dans la littérature montrent qu'ils surviennent le plus souvent au-delà de la cinquantaine [4, 5]. Le diagnostic est généralement tardif, devant des signes d'appels variés (leucorrhées, métrorragies, douleurs, prurit, saignement, palpation d'une masse) secondaires à l'envahissement de l'épithélium muqueux avec ulcération et surinfection [2]. Sur la plan clinique, la tumeur peut être uni ou multifocale, parfois pigmentée. Dans le mélanome vaginal, la tumeur siège le plus souvent au niveau du tiers inférieur (58%), et au niveau de la paroi antérieure (45%) [6] et c'est le cas de notre 2^ème^ observation.

Les mélanomes vulvaires siègent le plus souvent sur les petites lèvres et la face interne des grandes lèvres (de 50 à 65% des cas). La confirmation du diagnostic est histologique et fait appel à l'étude immunohistochimique. Il s'agit le plus souvent d'une prolifération cellulaire agencée en nappes où le pléomorphisme nucléaire est très marqué et l'index mitotique est élevé. Certaines cellules sont fusiformes, d'autres épithélioides ou pseudo-épithéliales avec parfois des cellules géantes plurinucléées. Le stroma est grêle réduit au réseau vasculaire. Les cellules tumorales peuvent contenir du pigment mélanique dans leur cytoplasme.

Les caractéristiques immuno-histochimiques sont similaires à celles des mélanomes cutanés. les marqueurs tumoraux utilisés pour affirmer le diagnostic, sont l'anticorps anti Melan A, anti protéine S100, et l'anticorps HMB45, ce dernier étant plus spécifique de la cellule mélanique.L'utilisation des deux anticorps est souhaitable. Plus récemment, d'autres anticorps plus sélectifs de la lignée mélanocytaire sont utilisés: le NK1 /C-3 et le NK1BETEB. Une fois le diagnostic d'un mélanome est porté, il reste à prouver sa nature primitive. Ceci implique la recherche d'un mélanome primitif cutané, ophtalmologique, ORL digestif ou d'antécédents d'exérèse antérieure d'une lésion cutanée pigmentaire. Les facteurs pronostics des mélanomes admis par l'AJCC révisé en 2009, notamment l'ulcération et le Breslow ne semblent pas utiles dans les mélanomes muqueux [7]. Cependant, après une revue de 115 cas de mélanomes vaginaux, Reid et al ont retenu la taille comme seul facteur pronostic avec une valeur seuil de 3cm [8]. La plupart des auteurs tels que Mordel et al, défendent l'utilisation du système de FIGO (International Federation Of Gynecologists And Obstetricians) [9] pour le staging, vue que le mélanome cervical est souvent diagnostiqué à un stade tardif et a une présentation clinique et un mode d'extension tout à fait comparable au carcinome cervical.

Comme dans les mélanomes cutanés, la chirurgie est aujourd'hui encore le seul traitement potentiellement curatif de cette tumeur. Geisler et al suggèrent une exentération pelvienne quand l'invasion dépasse 3mm [10]. Chung et Ariel quant à eux optent pour une chirurgie radicale complétée par un curage régional [11]. Dans le mélanome cervical, l'hystérectomie totale avec annexectomie bilatérale est la technique la plus utilisée. L'apport de la radiothérapie n'est pas admis par tous les auteurs. Le traitement adjuvant systémique du mélanome est décevant. L'immunothérapie par l'interféron n'augmente pas la survie globale et est associée à une toxicité importante. Au stade métastatique, le traitement du mélanome était jusqu'à présent palliatif faisant appel à une chimiothérapie à base de Dacarbazine (Deticène) avec des taux de réponse rapportés modestes sans aucune preuve d'allongement significatif de la survie. Aujourd'hui depuis un plus d'an le paysage thérapeutique du mélanome se modifie avec l'avènement de thérapies ciblées anti BRAF ou anti kit pour les mélanomes porteurs d'une de ces mutations avec immunothérapie par l'anticorps anti CTLA4 (Ipilimumab). Les mutations de BRAF sont retrouvées dans 50% des cas de mélanome cutanés mais sont rares dans les mélanomes vaginaux. En revanche ces derniers sont porteurs dans 10 à 15% de mutation KIT [12] et c'est le cas de notre 2^ème^ observation qui a bénéficié d'un traitement pas les inhibiteurs de Kit (imatinib) avec régression des nodules pulmonaires et de la masse après trois cures d'imatinib.

Malgré les différents types de traitements proposés, le pronostic des mélanomes vaginaux et cervicaux reste réservé avec un taux de survie à 5ans de 5 à 10% [13] pour les mélanomes vaginaux et de 0 à 18, 8% pour les mélanomes du col utérin. Le pronostic des mélanomes vulvaires dans les séries rapportées est aussi défavorable en raison de l′épaisseur élevée de la tumeur lors du diagnostic et la survie moyenne à 5 ans est estimée de 30 à 60% dans les dernières séries [14]. Ceci est dû au diagnostic qui se fait à un stade tardif mais aussi à la richesse du réseau vasculaire et lymphatique au niveau du vagin et de la vulve [7].

## Conclusion

Le mélanome de la muqueuse génitale féminine est une pathologie rare. Le diagnostic se fait le plus souvent à un stade tardif. Le pronostic est péjoratif marqué par des récidives fréquents et précoces. Plusieurs thérapeutiques sont proposées notamment en cas métastases avec des résultats encourageants.
